# The prevalence of undernutrition among students attending traditional Ethiopian orthodox Tewahedo church schools in northwest Ethiopia

**DOI:** 10.3389/fpubh.2023.1124173

**Published:** 2023-07-03

**Authors:** Yalelet Fentaw Shiferaw, Desale Bihonegn Asmamaw, Melaku Tadege Engidaw, Daniel Gashaneh Belay, Haileyesus Birhan, Wubshet Debebe Negash

**Affiliations:** ^1^Department of Nutritional Care and Counseling, University of Gondar Specialized Hospital, Gondar, Ethiopia; ^2^Department of Reproductive Health, College of Medicine and Health Sciences, Institute of Public Health, University of Gondar, Gondar, Ethiopia; ^3^Department of Public Health (Human Nutrition), College of Health Sciences, Debre Tabor University, Debre Tabor, Ethiopia; ^4^Department of Human Anatomy, College of Medicine and Health Sciences, University of Gondar, Gondar, Ethiopia; ^5^Department of Epidemiology and Biostatistics, College of Medicine and Health Sciences, Institute of Public Health, University of Gondar, Gondar, Ethiopia; ^6^Health and Nutrition Senior Program Officer from Concern World Wide Ethiopia, Addis Ababa, Ethiopia; ^7^Department of Health Systems and Policy, College of Medicine and Health Sciences, Institute of Public Health, University of Gondar, Gondar, Ethiopia

**Keywords:** undernutrition, Ethiopia, adolescent males, orthodox, Gondar

## Abstract

**Background:**

Undernutrition is a major public health concern affecting the health, growth, development, and academic performance of adolescents studying in school. During this crucial period, dietary patterns have a vital impact on lifetime nutritional status and health. The problem of undernutrition among particular groups of adolescents attending traditional schools has not previously been studied. Therefore, this study aimed to assess the prevalence of undernutrition and associated factors among adolescents aged 10–19 years attending Orthodox Church schools in northwest Ethiopia.

**Methods:**

An institution-based, cross-sectional study design was employed, with data collected from March 1 to 30, 2021. A simple random sampling technique was used to recruit a total of 848 male attendees of traditional schools. Data were collected via an interviewer-administered semi-structured questionnaire. The nutritional status of participants was assessed using anthropometric measurements. The WHO Anthroplus software was used for analysis. Both bivariable and multivariable logistic regression analyses were conducted to identify the factors associated with nutritional status. The degree of association between the independent variables and the dependent variable was assessed using odds ratios, reported with 95% confidence intervals, and a threshold of *p* ≤ 0.05.

**Results:**

The prevalence of undernutrition was found to be 61.3% [95% CI: 58.1, 64.6]. The likelihood of developing undernutrition was elevated among those adolescents who were following the traditional school levels of *dikuna* (AOR = 4.3, 95% CI = 1.3, 13.6), *kinne* (AOR = 4.5, 95% CI = 1.4, 14.6), *aquaquame* (AOR = 9.9, 95% CI = 2.5, 39.88), *tirguame* (AOR = 6.4, 95% CI = 1.6, 25.6), and among those whose mothers had no formal education [AOR = 3.7, 95% CI: 1.2, 12.8]. In contrast, those adolescents who always washed their hands after a toilet visit had lower odds of undernutrition than their counterparts [AOR = 0.7, 95%CI: 0.5, 0.98].

**Conclusion:**

More than three out of five participating male adolescents were undernourished. Thus, to improve the nutritional status of adolescents studying in traditional church schools, extensive health education for these adolescents is essential. Moreover, the establishment of well-resourced traditional religious school, equipped for the provision of an adequate, diversified diet, is important. Developing the habit of handwashing after visiting the toilet and before and after food preparation is also recommended for adolescent students.

## Background

Adolescence is the transition period between childhood and adulthood, encompassing people aged 10–19 years (1). Adolescents account for a disproportionately large percentage of the population in developing countries (2). There are 1.2 billion adolescents aged 10–19 in the world, forming 18% of the world’s population (3). In Africa, the population of adolescents accounts for more than 265 million people (4).

Growth is faster during adolescence than at any other time; this in turn increases nutritional needs at this juncture, as adolescents gain up to 50% of their adult weight, more than 20% of their adult height, and 50% of their adult skeletal mass during this period (5). Adolescents are a nutritionally vulnerable group for a number of specific reasons, including their high requirements for growth, their eating patterns and lifestyles, their risk-taking behaviors, and their susceptibility to environmental influences (6). Inadequate nutrition in adolescence can potentially retard growth and sexual maturation, although these are also likely consequences of chronic malnutrition in early infancy and childhood (6). Short stature in adolescents resulting from chronic undernutrition is associated with reduced lean body mass and deficiencies in muscular strength and working capacity (7, 8).

Studies in Ethiopia on the magnitude of the problem of undernutrition among female adolescents have reported rates ranging from 14.4% in Gondar (9) to 19.5% in the town of Dessie (10). In addition, 29.2% of adolescents have been reported to be stunted, and 30.4% to exhibit wasting (11, 12). Systematic reviews have also revealed that the rates of stunting and underweight among adolescent girls are 20.7 and 27.5%, respectively (13). Furthermore, a recent demographic study has revealed that the prevalence of undernutrition among adolescent girls and young women is 25% (14). Factors such as large family size, occupation, diet-related knowledge, and monthly *per capita* income are contributors to adolescent malnutrition (15, 16). Moreover, rural residence, an unprotected source of drinking water, a lack of latrine, low dietary diversity score, and mother illiteracy have been identified as contributing factors for adolescent undernutrition (13, 17).

Teaching activities conducted by the Ethiopian Orthodox Church has been relaying traditional teachings and schools of thoughts for centuries; these teachings form a deeply embedded component of religious education, life, and ethos in the Ethiopian Orthodox Church. The types of teaching provided include church singing and movement (*aquaquame*), poetry (*kinne*), and commentaries on the Bible and on the writings of the Church fathers and the monks. In addition to the many centuries of church teachings, traditional Ethiopian Orthodox education has been a deeply embedded component of education, culture, and social activities across the country (18). However, the life of students undergoing traditional teaching is hard and demanding for contemporary boys. For example, they leave their family; they beg for their food among families of the neighborhood, but also sometimes far away; and most of the time, their daily life as students within the church tradition is one of poverty and is very demanding (19).

Religious youths are less likely than other youths to engage in behaviors that are potentially detrimental to their health (20). In terms of the consumption habits of church followers in Ethiopia, religious culture here places a total taboo on the use of animal products such as milk and butter during fasting (21).

Although a great deal of research has been carried out to understand the nutritional status of formally enrolled school students during the period of adolescence in Ethiopia (22–25), to the best of the investigators’ knowledge, there has been no attempt to understand the nutritional status of male adolescents studying in the Ethiopian Orthodox Tewahedo Church. The neglect of this topic indicates that students receiving education in the setting of traditional schools have been ignored by research efforts. Although nutritional studies have been conducted in modern schools, it is also crucial to investigate the magnitude of the problem of undernutrition among adolescents in these traditional schools. Therefore, the aim of this study was to assess the prevalence of undernutrition and its associated factors among attendees of Ethiopian Orthodox Tewahedo Church traditional schools. The findings should alert policymakers, program developers, and healthcare workers to the need for appropriate and science-based interventions, such as boarding arrangements including the adequate provision of school food, would reduce the burden on students of searching for food in the community. Additionally, the results will serve as a baseline for future researchers within the broad area of such religious settings.

**Figure 1:**
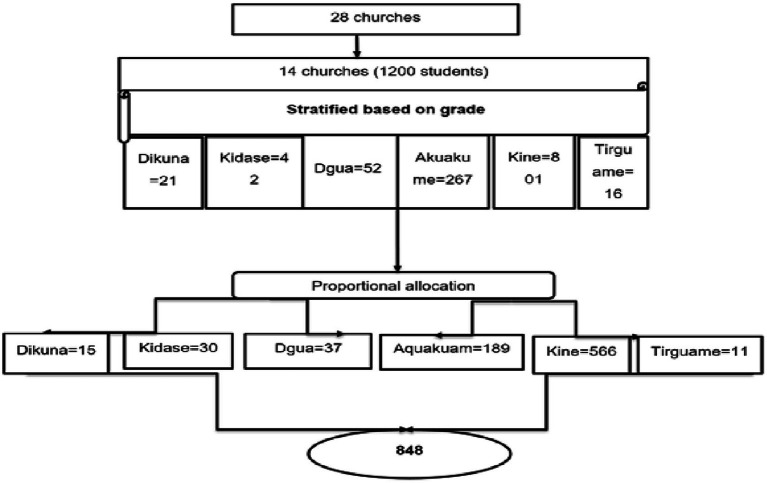
Schematic presentation of the sampling procedure for recruitment of adolescents in northwest Ethiopia, 2021.

## Methods

### Study design, area, and period

An institution-based cross-sectional study was conducted in Gondar city, northwest Ethiopia, from March 1 to 30, 2021. The institutions included in the study were specifically the orthodox churches. Gondar is one of the ancient cities and is located 728 km from Addis Ababa in northwest Ethiopia. The city has 28 orthodox churches with schools, and there are approximately 3,500 male adolescents studying at the traditional schools across these 28 churches (Figure 1).

### Population

The source population for this study consisted of all male students enrolled in traditional schools who were followers of the Ethiopian Orthodox Tewahedo Church in Gondar city. Almost all of these students are adolescents (aged 10–19 years). Those male adolescents who were available during the study period were included in the study population.

### Sample size and sampling procedure

The required sample size for examining undernutrition was determined using the formula for a single population proportion with the following assumptions:

*p* = 50% (this value was taken due to a lack of previous research among traditional school students aged 10–19 years).

*Z* (level of significance) = 95%.

*d* (margin of error) = 5%.


$$n=\frac{{\left(Z\alpha /2\right)}^{²}\;P\;\left(1-P\right)}{d^{²}}$$

$$n=\frac{{\left(1.96\right)}^{²}\;0.5\;\left(1-0.5\right)}{{\left(0.05\right)}^{²}}$$

$$n=\frac{{\left(1.96\right)}^{2}\;\left(0.5\right)\;\left(0.5\right)}{{\left(0.05\right)}^{²}}=384.5\sim 385$$

Adding a 10% non-response rate gave a total required sample size of 424; considering the design effect of accommodating two different levels of education, the final sample size required for the study was 848.

## Study variables and measures

The outcome variable of this study was the nutritional status of male adolescents who had attended a traditional school in the Ethiopian Orthodox Tewahedo Church. Nutritional status was classified based on the World Health Organization (WHO) BMI-for-age classification of malnutrition in adolescents, under which a *Z* score < −3 represents severe malnutrition, *Z* > −3 and < −2 represents moderate malnutrition, *Z* > −2 < −1 represents mild malnutrition, and Z > −1 and < 1 score is normal nutrition status.

In addition to this outcome variable, the independent variables measured in this study were: sociodemographic factors (age, household income, educational status, and occupation); nutrition-related factors (hygiene, eating habits, eating pattern); environmental factors (sanitation, housing conditions, latrine access, water source); and other factors, such as social support, duration of fasting, alcohol consumption, food eating practices, history of diarrheal illness, vomiting, food sources, availability of storage for a variety of food, and number of students per house.

### Operational definitions

#### Traditional school attendees

For this study, traditional school attendees were considered to be those students of the Ethiopian Orthodox Tewahedo Church schools whose age fell within the range of 10–19 years. The aspects of traditional education within these schools were classified as follows: *dikuna* (baseline and first level of education), *kinne* (church poetry), *aquaquame* (lessons on church hymns of differing complexity), *dgua* (the book of songs), *tirguame* (interpretation of church books), and *kidase* (traditional chanting) (26, 27).

#### Fasting

Fasting was considered to entail abstaining from all food for 13 h (mid night to 1 pm during the daytime) within a 24-h period.

#### Diarrhea

Those who had three or more episodes of diarrhea in the 24 h prior to the survey were considered to have diarrhea.

#### Alcohol consumption

For this study, adolescents were considered to consume alcohol if they drank alcohol (tella, arekie, or beer) any number of times per week; alcohol consumption was coded as 1 = yes and otherwise 0 = no.

#### Amount of food eaten per sitting

This was assessed based on whether the respondent reported eating until satiety or not.

#### Frequency of eating breakfast

The frequency of eating breakfast was classified as sometimes (only on Saturday and Sunday), often (on Monday, Tuesday, Thursday, Saturday, and Sunday), or always (every day of the week).

### Data collection tools and procedures

Prior to collection of any data, official permission was obtained from North Gondar Church Administration Office and from the director of each traditional church school; sampling units (adolescent students) were then identified and designated with their own code corresponding to their church. Eight graduate students of health science were provided with training and were responsible for the data collection and supervisory processes for all 28 churches. The primary investigator provided 2 days of training for the data collectors and supervisors on how to conduct anthropometric measurements, the MUAC, and interview techniques. A data collection manual was prepared and provided to the data collectors and supervisors during the period of data collection. The data were collected by the trained data collectors using a face-to-face interview technique employed a coded, structured questionnaire.

The nutritional status of study participants was determined using the Measure Anthrop software package based on weight and height measurements taken according to standard procedures described by the WHO. Weight was measured to the nearest 0.1 kg using a Seca electronic scale with the adolescent wearing a uniform or light clothing and without shoes. Weight was recorded twice and the mean value was used in the analyses. The scale was checked for accuracy with known standard weights (e.g., 2 kg) after every measurement. A wooden stand placed on a flat surface was used to measure each individual’s height. The subject stood on the base of the device with feet together (without shoes). The shoulders, buttocks, and heels were required to touch the vertical measuring board. Height was measured to the nearest 0.1 cm; it was recorded twice, and the mean value was calculated, recorded, and entered into the analysis. Computed Z-scores of Body Mass Index for age (BMIAZ) and height for age (HAZ) were used to assess thinness and stunting, respectively.

### Data quality control

The questionnaire was first prepared in English and then translated to the local language (Amharic) by an expert and back-translated into English. The aim of the study was explained to the church leaders and adolescents. The quality of the data was assured by the use of a properly designed and pre-tested questionnaire and through standard and appropriate supervision of the data collectors. Overall supervision was provided by the principal investigator. For standardization of the questionnaire, a pre-test of the questionnaire and anthropometric measurements was performed at one church that was not part of the study. An appropriate modification was made after analyzing the pre-test results before proceeding with data collection. Completed questionnaires were reviewed and checked for completeness and relevance by the supervisors and principal investigator, and any necessary feedback was offered to the data collectors within a few days.

### Data processing and analysis

All the returned questionnaires were checked manually. The data were cleaned before being coded and entered into WHO Anthro Plus, Epi Info version 7, and SPSS software version 20. The collected data were cleaned and checked again, descriptive statistics were computed, and bivariate and multivariate analyses were conducted. Body mass index (BMI) was computed as weight (kg) per meter of height squared (m^2^) (kg/m^2^). The 2007 WHO growth reference was used as a standard reference during analysis. Crude and adjusted ORs, with their associated 95% CIs, were computed to assess the presence of associations between the explanatory variables and the outcome variable. Multicollinearity was assessed, and the data were found to contain no multicollinearity (mean VIF = 2.14). The Hosmer–Lemeshow test was used to test for model fit; the goodness of fit was found to be *p* = 0.51, which is appropriate for logistic regression models.

## Results

### Sociodemographic characteristics of the participants

A total of 848 male adolescent respondents were included in the study. The majority of the respondents (705; 83.1%) were in late adolescence. Regarding the formal educational status of the students, 370 (43.6%) were able to read and write and 421 (49.6%) had completed their primary education. Regarding their level of traditional education, 566 (66.7%) were studying at the *kinne* level and 189 (22.3%) were studying *aquaquame*.

The main source of food (for 814 respondents; 96%) was the community. Among all study participants, 757 (89.3%) reported not washing their hands after visiting the toilet. The majority of the participants (65.1%) had a history of illness (Table 1).

**Table 1:** Sociodemographic characteristics and eating/fasting-related habits of male adolescents studying in traditional schools of the Orthodox Tewahedo Church, northwest Ethiopia, 2021.

Characteristics	Number	Percentage (%)
Age group (years)		
10–14	143	16.9
15–19	705	83.1
Educational status		
Able to read and write	285	33.6
Primary education	473	55.8
Secondary education and above	90	10.6
Level of study in traditional education		
Dikuna	15	1.8
Kinne	566	66.7
Aquaquame	189	22.3
Duga	37	4.4
Truguame	11	1.3
Kidase	30	3.5
Father’s educational status		
Unable to read and write	370	43.6
Able to read and write	421	49.6
Primary education and above	57	6.7
Mother’s educational status		
Unable to read and write	615	72.5
Able to read and write	220	25.9
Primary education and above	3	1.5
Alcohol consumption		
Yes	715	84.3
No	133	15.7
Breakfast on non-fasting days		
Yes	788	92.9
No	60	7.1
Frequency of eating breakfast on non-fasting days		
Sometimes	488	57.6
Often	308	36.3
Always	52	6.1
Type of food eaten		
Bread	15	1.8
Injera	833	98.2
Frequency of eating per day		
Once	17	2.0
Twice	480	56.6
Three times	337	39.7
Four times	14	1.7
Type of food obtained from the community		
Fresh food	323	37.7
Leftover food	525	61.3
Amount of food eaten per sitting		
To satiety	614	72.4
Not to satiety	234	27.6
Main source of food		
Church	16	1.9
Family	18	2.1
Community	814	96.0
Sufficient food obtained from the community		
Yes	672	79.2
No	176	20.8
Storing food		
Yes	813	95.9
No	35	4.1
Storage container for prepared food		
Open container	598	70.5
Covered container	250	29.5
Duration of food storage		
Less than one day	453	53.4
One or two days	316	37.3
Three days or more	79	9.3
Food preservation methods		
Sun-drying	813	95.9
Adding salt	35	4.5
Methods of water storage		
Covered containers	571	67.3
Open containers	277	32.7
Washing hands before eating		
Yes	839	98.93
No	9	1.17
Washing hands after eating		
Yes	757	89.27
No	91	10.73
Washing hands before and after eating		
Yes	726	85.6
No	122	14.4
Washing hands after visiting the toilet		
Yes	91	10.7
No	757	89.3
Method of washing hands		
Using water only	672	79.2
Sometimes using soap	166	19.6
Always using soap	10	1.2
Number of students per room		
3–5	532	62.7
6–8	312	36.8
9–12	4	0.5
History of illness in the past month		
Yes	552	65.1
No	296	34.9
Source of money for daily use		
Family	160	18.9
Daily labor	624	73.6
None	64	7.5
Support from family with food and money		
Yes	265	31.3
No	583	68.8

### Physical activity and frequency of consumption of food groups

In terms of physical activity, the majority (99.4%) of respondents reported engaging in work involving moderate-to-vigorous activity, and 333 (39.3%) reported working 7 times per week. In their responses to a 24-h recall history of eating (Table 2), none of the respondents (0%) reported having consumed fresh meat, organ meat, eggs, or milk within the last 24 h.

**Table 2:** Physical activity and frequency of consumption of food groups among adolescents studying in traditional schools, Gondar, 2021.

Variable	Number	Percentage (%)
Work: moderate-to-vigorous activity		
Yes	843	99.4
No	5	0.6
Frequency of work per week		
3–5 times	825	97.3%
6–7 times	23	2.7%
Engaging in vigorous sporting activity		
Yes	838	98.8
No	10	1.2
Time taken to collect food from the community		
3–5 h	343	40.4
6–8 h	505	59.6
**24-h recall questionnaire on eating habits: foods eaten yesterday**		
Starchy staple		
Yes	701	82.7
No	147	17.3
Dark green leafy vegetables		
Yes	619	73.0
No	229	27.0
Other fruits and vegetables rich in Vitamin A		
Yes	591	69.7
No	257	30.3
Other fruits		
Yes	634	74.8
No	214	25.2
Legumes/nuts		
Yes	624	73.6
No	224	26.4

### Prevalence of undernutrition

**Figure 2:**
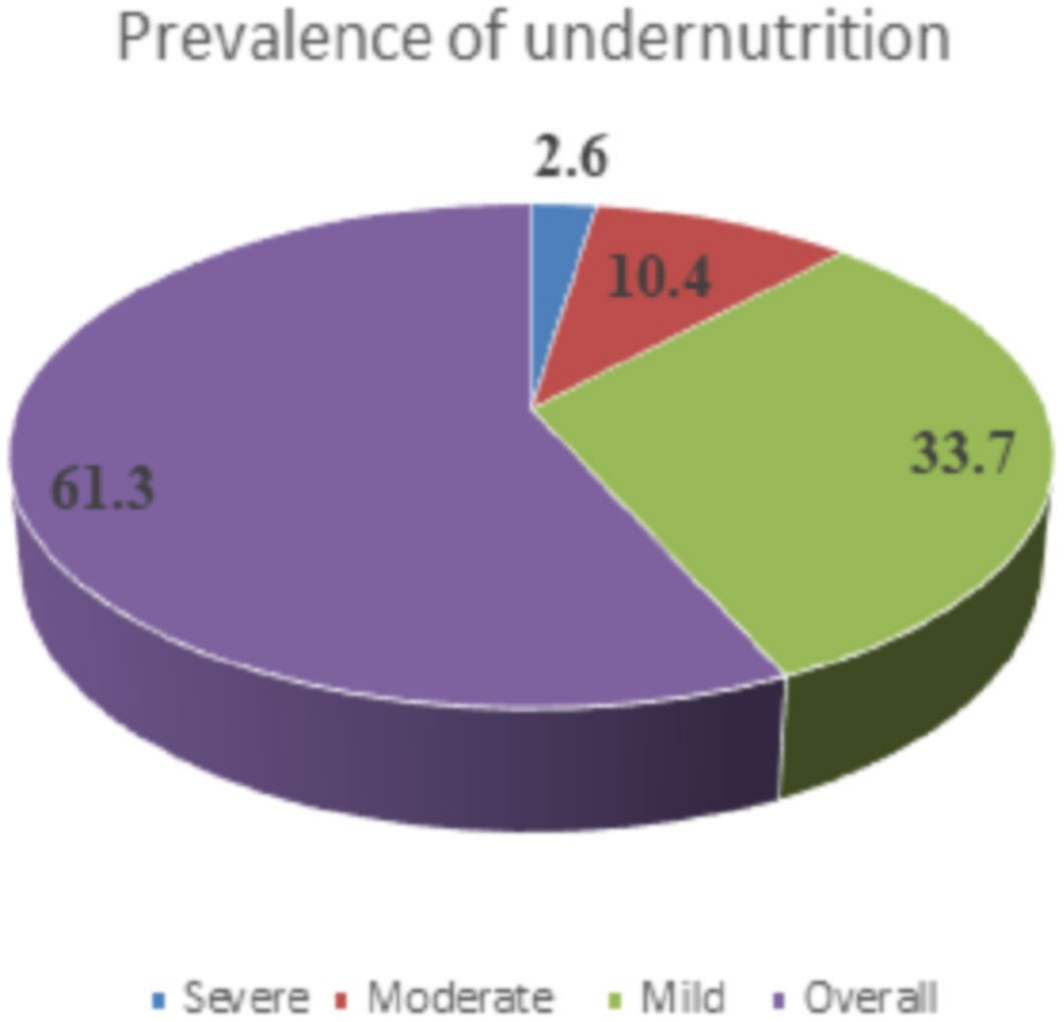
Prevalence of undernutrition among male adolescents attending traditional schools in northwest Ethiopia, 2021.

Based on anthropometric measurement, the prevalence of undernutrition among the adolescents was found to be 61.3% (95% CI: 58.1, 64.6) (Figure 2).

### Factors associated with undernutrition among male adolescents

Although the data were ordinal in nature, the proportional odds assumption was not found to be reasonable (*p* ≤ 0.001). Consequently, a binary logistic regression analysis was conducted to assess the association of various factors with undernutrition. Variables with a *p-*value < 0.25 in the binary logistic regression analysis were entered into a multivariate logistic regression and associated factors were thus identified.

Compared to students of the *kidase* level, the odds of undernutrition among *dikuna*, *kinne*, *aquaquame*, and *tirguame* students were 4.3 (AOR = 4.3, 95% CI = 1.3, 13.6), 4.5 (AOR = 4.5, 95% CI = 1.4, 14.6), 10 (AOR = 9.9, 95% CI = 2.5, 39.88), and 6.4 (AOR = 6.4, 95% CI = 1.6, 25.6) times higher, respectively.

The odds of undernutrition were higher among those adolescents who were not able to read and write than among those who were (AOR = 3.7, 95% CI = 1.13, 12.8).

The odds of undernutrition were 0.7 times lower among those adolescents who practiced handwashing before and after eating (AOR = 0.7, 95% CI = 0.50, 0.98). Those adolescents who reported washing their hands after visiting the toilet were 0.7 times less likely to meet the criteria for undernutrition (AOR = 0.7, 95% CI = 0.47, 0.96) as compared to their counterparts (Table 3).

**Table 3:** Results of a multivariate logistic regression assessing factors associated with undernutrition among adolescents at traditional schools in Gondar, Ethiopia, 2021.

Variables	Undernutrition		COR (95% CI)	AOR (95% CI)	*p*
	Yes	No			
Age (years)					
Early adolescence (10–14)	78	65	1.4 (0.97–2.013)	1.5 (0.99–2.23)	0.77
Late adolescence (15–19)	442	263	1	1	
Educational status					
Able to read and write	173	112	1.1 (0.7–1.7)	1.5 (0.99–1.3)	0.15
Primary education	294	179	1.1 (0.7–1.8)	1.2 (0.65–1.9)	0.98
Secondary and above	53	37	1	1	
Level of study in traditional education					
Dikuna	4	11	0.16 (0.04–0.62)	**4.3 (1.3–13.6)	0.01
Kinne	344	222	0.7 (0.3–1.5)	**4.5 (1.4–14.6)	0.01
Aqaqame	117	72	0.7 (0.3–1.6)	**9.9 (2.5–39.88)	0.001
Digua	29	8	1.6 (0.5–4.7)	2.3 (0.4–11.9)	0.19
Turigame	5	6	0.4 (0.9–1.5)	**6.4 (1.6–25.6)	0.01
Kidase	21	9	1	1	
Father’s educational status					
Unable to read and write	222	148	0.5 (0.3–1)	0.5 (0.3–1.001)	0.93
Able to read and write	256	156	0.6 (0.3–1)	0.6 (0.23–1.03)	0.51
Primary education and above	42	15	1	1	
Mother’s educational status					
Unable to read and write	372	243	2.45 (0.8–7.6)	**3.7 (1.13–12.8)	0.01
Able to read and write	143	77	2.97 (0.9–9.4)	3.4 (0.96–11.8)	0.64
Primary education and above	5	8	1	1	
Mother’s occupation					
Housewife	424	258	1.3 (0.8–2.04)	1.2 (0.7–2)	0.57
Merchant	53	37	1.09 (0.6–2.04)	1.2 (0.4–2.9)	0.52
Day-laborer	43	33	1	1	
Father’s occupation					
Farmer	415	273	0.6 (0.3–1.02)	0.6 (0.3–1.14)	0.64
Merchant	33	19	0.6 (0.3–1.4)	0.5 (0.2–1.34)	0.80
Government employee	17	15	0.4 (0.2–1.04)	0.37 (0.1–1.9)	0.59
Day-laborer	12	5	0.9 (0.3–2.9)	0.9 (0.3–3.17)	0.71
Private employee	43	16	1	1	
Alcohol consumption					
Yes	449	266	1.5 (1.01–2.14)	3.9 (0.46–1.12)	0.61
No	71	62	1	1	
Fasting					
Sometimes	298	190	0.9 (0.7–1.3)	0.8 (0.46–1.512)	0.67
Most of the time	188	120	1.2 (0.7–2.2)	0.8 (0.45–1.535)	0.52
Always	34	18	1	1	
Main source of food					
Church	50	39	0.8 (0.5–1.2)	0.8 (0.4–1.5)	0.81
Family	26	18	0.9 (0.5–1.6)	0.9 (0.35–1)	0.93
Community	444	271	1	1	
Storing food					
Yes	495	318	0.6 (0.3–1.3)	2.6 (0.104–1)	0.87
No	25	10	1	1	
Ate other fruits yesterday					
Yes	385	249	0.9 (0.7–1.3)	0.7 (0.5–2.23)	0.77
No	135	79	1	1	
Ate fruits and vegetables rich in Vitamin A yesterday					
Yes	376	215	0.9 (0.7–1.3)	0.3 (0.6–1)	0.62
No	144	113	1	1	
Access to latrine					
Yes	437	274	1.04 (0.7–1.5)	0.6 (0.05–6.3)	0.95
No	83	54	1	1	
Washing hands before and after eating					
Yes	448	278	1.12 (0.8–1.7)	**0.7 (0.5–0.98)	0.001
No	72	50	1	1	
Washing hands after visiting the toilet					
Yes	63	28	1.5 (1.05–2.12)	**0.69 (0.47–0.96)	0.012
No	457	300	1	1	
History of illness in the past month					
Yes	335	217	0.9 (0.7–1.2)	0.84 (0.6–1.15)	0.95
No	185	111	1	1	
Source of money for daily use					
Family	104	56	0.8 (0.6–1.2)	0.7 (0.3–1.24)	0.61
Daily labor	381	243	0.7 (0.4–1.2)	0.6 (1.2–1.8)	0.68
None	35	29	1	1	

AOR, adjusted odds ratio; COR, crude odds ratio; *significant at *p* < 0.05; **significant at *p* < 0.01.

## Discussion

In terms of the prevalence of undernutrition, the findings of this study revealed that more than three out of five adolescent students at traditional schools are undernourished. This implies that a large number of Ethiopian Orthodox Church students are undernourished.

Specifically, the findings revealed that the prevalence of undernutrition in Gondar is 61.3%. This figure is higher than the findings of other studies, which have reported a prevalence of 26.4% in southern Ethiopia (28) and 14.4% in Gondar (9). A possible reason for this difference might be the differences in study population, period, and sample size. The current study was conducted among male adolescents who were attending traditional schools, whereas all the aforementioned previous studies were conducted among adolescent girls. Additionally, almost all of these previous studies were conducted 5 years ago. The higher prevalence of undernutrition in this study also might be accounted for by the fact that most of these traditional school attendees were practicing fasting for approximately 200 days per year (practice that involves abstaining from all types of foods for a large portion of the day) and that these students ate by begging for their daily food from the community (29, 30). Therefore, nutritional programs and interventions must include these traditional schools in Ethiopia. Additionally, it might be very important to enhance nutritional awareness among Ethiopian Orthodox church leaders.

Those adolescent attendees of traditional schools who were following the levels of *aquaquame*, *Turguame*, *dikuna*, and *kinne* had higher odds of undernutrition than students who were following the level of *kidase*. This might be because those who are following *kidase* are most likely to be provided with the opportunity to eat after each of the ceremonial activities of *kidase* (29). An additional possibility might be that all the former level of educations are finally leading to employment for *kidase* students after their graduation. This implies that the establishment of boarding with the provision of a diversified diet by the churches could be an important intervention.

With regard to the mother’s educational status, the findings of this study revealed that the odds of undernutrition were higher among adolescents whose mothers were unable to read and write than among those adolescents whose mothers had received some education. This might be because education enables mothers to understand the effects of undernutrition (31). Therefore, extensive education on nutritional concepts for mothers might be a very important intervention to overcome the problem of undernutrition in these traditional schools.

Finally, the likelihood of undernutrition was higher among male adolescents who did not wash their hands before and after eating. Similarly, those who did not wash their hands after visiting the toilet had higher odds of undernutrition. This finding is similar to those of other studies conducted in Ethiopia (31, 32) and Iraq (33). Therefore, personal hygiene and sanitation measures, such as washing the hands with soap after each toilet visit and before eating food, are recommended for the students.

### Strengths and limitations of the study

The study had a large sample size and specifically targeted individuals attending traditional schools. On the other hand, because of the cross-sectional nature of the data, it is difficult to generalize the findings to the entire population and to identify the relationships between cause and effect.

## Conclusion

The finding of this study showed that the prevalence of undernutrition among traditional Orthodox Church students is high. Students at the levels of *aquaquame*, *Turguame*, *dikuna*, and *kinne* were at high risk of undernutrition. Moreover, not washing one’s hands before and after eating and after visiting the toilet was significantly associated with undernutrition among male adolescents. Therefore, to improve the nutritional status of the students, the establishment of well-resourced traditional religious schools, equipped for the provision of an adequate diversified diet, is important; additionally, parents should develop the habit of serving a good diversity of foods to their adolescents. Furthermore, encouraging students to wash their hands before and after eating as well as after visiting the toilet is very important. Finally, we recommend that further research should be conducted targeting these students at traditional schools across a wide area, taking both qualitative and quantitative approaches.

## Data availability statement

The raw data supporting the conclusions of this article will be made available by the authors, without undue reservation.

## Ethics statement

The studies involving human participants were reviewed and approved by Ethical clearance was obtained from the institutional review board of University of Gondar. Additionally, Institution of Ethiopian Orthodox Church (ETOC) North Gondar Sinod Branch formal letter of cooperation was written for Gondar city intuition of church and respective churches were also obtained. Informed consent was obtained from each study subject. Respondents were also obtained about the objective of study which contributed necessary information for policy makers and other concerned bodies. Brief explanation about the purpose of the study was given to each concerned bodies. The voluntary nature of the study was explained for the study participants. Any involvement in the study was done after their complete consent was obtained. They were also informed that all data obtained from them would be kept confidential by using codes instead of any personal identifiers. The patients/participants provided their written informed consent to participate in this study.

## Author contributions

YS and WN conceived and designed the research and performed the analysis. DA, ME, HB, and DB were involved in supervision and reviewed the manuscript. YS, DA, ME, DB, HB, and WN revised the manuscript, and WN subsequently revised the final draft of the article. All authors contributed to the article and approved the submitted version.

## Funding

The University of Gondar sponsored this study. However, it played no role in the decision-making relating to manuscript preparation or publication.

## Conflict of interest

The authors declare that the research was conducted in the absence of any commercial or financial relationships that could be construed as a potential conflict of interest.

## Publisher’s note

All claims expressed in this article are solely those of the authors and do not necessarily represent those of their affiliated organizations, or those of the publisher, the editors and the reviewers. Any product that may be evaluated in this article, or claim that may be made by its manufacturer, is not guaranteed or endorsed by the publisher.

## Abbreviations

AOR: Adjusted odds ratio
BMI: Body mass index
COR: Crude odds ratio
ETOC: Ethiopian Orthodox Church
MUAC: Mid-upper arm circumference
OR: Odds ratio
SPSS: Statistical package for the social sciences.
